# Relationship Between Maternal Pre-pregnancy Weight and Offspring Weight Strengthens as Children Develop: A Population-Based Retrospective Cohort Study

**DOI:** 10.2188/jea.JE20170137

**Published:** 2018-12-05

**Authors:** Yuki Fujita, Katsuyasu Kouda, Harunobu Nakamura, Masayuki Iki

**Affiliations:** 1 Department of Public Health, Kindai University Faculty of Medicine, Osaka, Japan; 2 Department of Health Promotion and Education, Graduate School of Human Development and Environment, Kobe University, Kobe, Japan

**Keywords:** growth, maternal weight, pediatric obesity, pregnancy

## Abstract

**Background:**

Maternal pre-pregnancy weight has been reported to be positively associated with offspring weight. The association between maternal weight and offspring weight might be explained by maternal lifestyle. We investigated the strength of the relationship between maternal body mass index (BMI) at the beginning of pregnancy and offspring BMI at several growth stages.

**Methods:**

The source population was all eighth graders registered in all public schools in the city of Fukuroi, Japan, in 2012. Records of maternal anthropometry at the beginning of pregnancy were obtained from the Maternal and Child Health (MCH) Handbook. The height and body weight of each student were measured. A regression model was used to assess the association between maternal BMI z-score at the beginning of pregnancy and offspring BMI z-score at various ages.

**Results:**

Of the source population, data from the MCH Handbook were obtained for 480 students. Among males, maternal BMI z-score was not associated with offspring BMI z-score at birth and at age 3 years but was associated with offspring BMI z-score at age 13 years (standardized regression coefficient (β) = 0.19; *P* < 0.01). Among females, maternal BMI z-score was associated with offspring BMI z-score at birth (β = 0.11; *P* < 0.05), at age 3 years (β = 0.22; *P* < 0.01) and at age 13 years (β = 0.51; *P* < 0.01).

**Conclusions:**

Our results suggest that the positive association between maternal weight at the beginning of pregnancy and offspring weight around puberty is stronger than that between maternal weight and offspring weight at birth. Maternal lifestyle may influence offspring weight in adolescence.

## INTRODUCTION

An overweight status, such as obesity, is an important risk factor for non-communicable diseases (NCDs), such as diabetes, stroke, coronary heart disease, and hypertension.^1^ As being overweight in childhood and adolescence leads to an overweight status in adulthood,^2^ prevention of overweight status in childhood could prevent NCDs later in life. There is a growing body of literature suggesting that factors related to the intrauterine nutritional environment, such as maternal body weight during pregnancy, may influence body weight in childhood and adolescence.^3^^,^^4^ A number of studies have reported that large gestational weight gain is a potential risk factor for childhood obesity.^5^^–^^7^

In addition to the effect of the intrauterine nutritional environment, body weight in childhood is known to be affected by childhood lifestyle habits, such as diet and physical activity. Young children are exposed to a variety of postnatal environmental variables, including maternal lifestyle. Maternal eating behavior has been reported to affect offspring eating behavior,^8^ whereas maternal pre-pregnancy weight is reportedly positively associated with offspring weight.^9^^–^^14^ However, the strength of the relationship between maternal weight and offspring weight differs among previous reports.^9^^–^^14^ With regard to relationships between maternal weight and offspring growth patterns, children born to mothers with overweight were reported to show a greater increase in body weight with age.^15^^–^^19^ However, there is still a shortage of knowledge about the strength of the association between maternal weight and offspring weight. In the present study, we assessed the relationship between maternal body mass index z-score at the beginning of pregnancy (mBMI-bp) and offspring BMI z-scores at several growth stages (birth, age 3 years, and age 13 years) in a population-based retrospective cohort study and revealed temporal characteristics of the relationship.

## METHODS

### Study participants

The source population consisted of all eighth graders (age 13–14 years; 803 students; 437 boys and 366 girls) registered in all public schools in the city of Fukuroi, Japan, in 2012. Almost all eighth graders in the city participated, because Fukuroi has no private junior high school. All participants received printed information regarding the study prior to their participation. Participants were allowed to decline participation on their own accord. The present study was approved by the Ethics Committee of Kindai University Faculty of Medicine.

### Health examination and measurements

In all public schools of Fukuroi, a health examination is conducted by The Fukuroi Board of Education for eighth graders from April through June. Licensed teachers measured the height and body weight of each student in accordance with methods set forth in the Japanese School Health Law. Height was measured to an accuracy of 0.1 cm and weight to 0.1 kg. BMI was calculated as weight divided by height squared (kg/m^2^).

As part of the health examination, parents or guardians of the participants transcribed information from the Japanese Maternal and Child Health (MCH) Handbook to a questionnaire of their own free will. The transcribed information included offspring body weight and length at birth, body weight and height at age 3 years, maternal height, maternal weight recorded at the beginning of pregnancy, the last maternal weight recorded before delivery, gestational age, maternal age at delivery, and smoking during pregnancy. In Japan, as part of MCH Services, one MCH Handbook is distributed to one child by the Government of Japan when a new pregnancy is registered. The MCH Handbook consists of records of antenatal care, including maternal weight and height, postpartum care, newborn and child care, and immunizations.^20^ Obstetricians, pediatricians, public health nurses, and midwives fill in the MCH Handbook at hospitals, clinics, or health centers at each event.^21^ BMI cut-offs for maternal overweight status and underweight status were 25 kg/m^2^ and 18.5 kg/m^2^, respectively. Overweight status was determined using the cut-off point of the International Obesity Task Force.^22^

### Statistical analysis

Statistical analysis was performed with SAS software for Windows, ver. 9.4 (SAS Institute Inc., Cary, NC, USA). The level of significance was set at *P* < 0.05. The unpaired *t* test was used to compare anthropometric characteristics of included participants with those of excluded participants and to compare anthropometric characteristics between males and females. The chi-square test was used to estimate independent proportions of underweight, overweight, and smoking. Pearson’s correlation coefficients were used to measure the strength of linear associations between maternal/offspring factors and mBMI-bp or offspring BMI z-scores at various ages.

Multiple regression analysis was used to test associations between offspring BMI z-score and maternal BMI z-score and gestational weight gain after stratification into tertiles by maternal BMI z-score and gestational weight gain. Multiple regression models were also used to evaluate associations between maternal BMI z-score and offspring BMI z-scores at several growth stages after adjusting for gestational age, maternal age at delivery, smoking during pregnancy, gestational weight gain, and offspring birth weight as potential confounders.^23^^–^^25^

## RESULTS

Among the 790 students who participated in the health examination, MCH Handbook records were obtained for 480 (262 males and 218 females). Table 1 summarizes the characteristics of the included and excluded participants, which did not significantly differ.

**Table 1.** Characteristics of participants included in and excluded from analysis

	Included participants (*N* = 480)	Excluded participants (*N* = 310)	*P* value
Male, *N* (%)	262 (54.6)	172 (55.5)	0.826
Height, cm	156.5 (7.0)	156.6 (7.3)	0.910
Weight, kg	47.2 (8.5)	48.0 (8.8)	0.189
BMI, kg/m^2^	19.2 (2.8)	19.5 (3.1)	0.123
Underweight^a^, *N* (%)	47 (9.8)	21 (6.8)	0.154
Overweight^a^, *N* (%)	42 (8.8)	36 (11.6)	0.222

BMI, body mass index; *N*, number.

Values represent mean (standard deviation) or *N* (percentage).

^a^The international cut-offs for body mass index were used to determine overweight and underweight children.

Table 2 shows basic characteristics of participants classified by offspring gender. There were no remarkable gender differences in maternal characteristics at the beginning of pregnancy, or in BMI and overweight prevalence of offspring at age 13 years. Height and weight in male offspring were greater than that in female offspring. Proportion of underweight offspring calculated using BMI was greater among female offspring than male offspring.

**Table 2.** Characteristics of participants by offspring gender

	Male (*N* = 262)	Female (*N* = 218)	*P* value
Maternal characteristics at the beginning pregnancy			
Age, years	28.2 (4.6)	27.5 (4.1)	0.075
BMI, kg/m^2^	20.6 (2.5)	20.1 (2.4)	0.066
Underweight^a^, *N* (%)	52 (19.8)	55 (25.2)	0.186
Overweight^a^, *N* (%)	18 (6.9)	10 (4.6)	0.332
Smoking, *N* (%)	13 (5.0)	13 (6.0)	0.688
Offspring characteristics at birth			
Gestational age, weeks	38.9 (1.7)	38.9 (1.7)	0.809
Birth weight, g	3,049.5 (421.0)	2,882.5 (413.5)	<0.001
Offspring characteristics at age 13 years			
Height, cm	158.5 (7.8)	154.1 (5.0)	<0.001
Weight, kg	48.2 (9.4)	45.9 (7.2)	0.004
BMI, kg/m^2^	19.1 (2.7)	19.3 (2.9)	0.342
Underweight^b^, *N* (%)	19 (7.3)	28 (12.8)	0.045
Overweight^b^, *N* (%)	29 (11.1)	13 (6.0)	0.053

BMI, body mass index; *N*, number.

Values represent mean ± standard deviation, or *N* (percentage).

^a^BMI cut-offs for maternal overweight status and underweight status were 25 kg/m^2^ and 18.5 kg/m^2^, respectively.

^b^International cut-offs for body mass index were used to determine overweight and underweight children.^22^

Table 3 shows relationship between maternal/offspring factors and mBMI-bp or offspring BMI z-scores at various ages. Maternal age at delivery was associated with mBMI-bp in both male and female offspring. Gestational age was associated with offspring BMI z-score at birth in male offspring and to offspring BMI z-scores at birth and at age 3 years in female offspring. Offspring birth weight was associated with mBMI-bp and offspring BMI z-scores at various ages in male offspring and to offspring BMI z-scores at birth and at age 3 years in female offspring. Gestational weight gain was associated with mBMI-bp in both male and female offspring.

**Table 3.** Relationship between maternal/offspring factors and mBMI-bp or offspring BMI z-score at various ages

maternal/offspring factor	mBMI-bp		Offspring BMI z-score at birth		Offspring BMI z-score at age 3 years		Offspring BMI z-score at age 13 years	
			
	*r*	*P* value	*r*	*P* value	*r*	*P* value	*r*	*P* value
Male								
Maternal age at delivery	0.186	0.003	0.042	0.498	−0.012	0.851	−0.057	0.356
Gestational age	−0.026	0.675	0.151	0.015	0.053	0.399	0.061	0.324
Offspring birth weight	0.129	0.037	0.386	<0.001	0.149	0.016	0.127	0.040
Gestational weight gain	−0.351	<0.001	0.073	0.243	0.074	0.232	0.098	0.113
Female								
Maternal age at delivery	0.136	0.045	0.105	0.121	0.101	0.139	−0.015	0.826
Gestational age	0.064	0.344	0.437	<0.001	0.188	0.006	0.024	0.729
Offspring birth weight	0.045	0.511	0.772	<0.001	0.324	<0.001	0.025	0.709
Gestational weight gain	−0.343	<0.001	0.051	0.456	0.025	0.719	−0.111	0.102

BMI, body mass index; mBMI-bp, maternal BMI z-score at the beginning of pregnancy; *r*, correlation coefficient.

**Figure 1.**
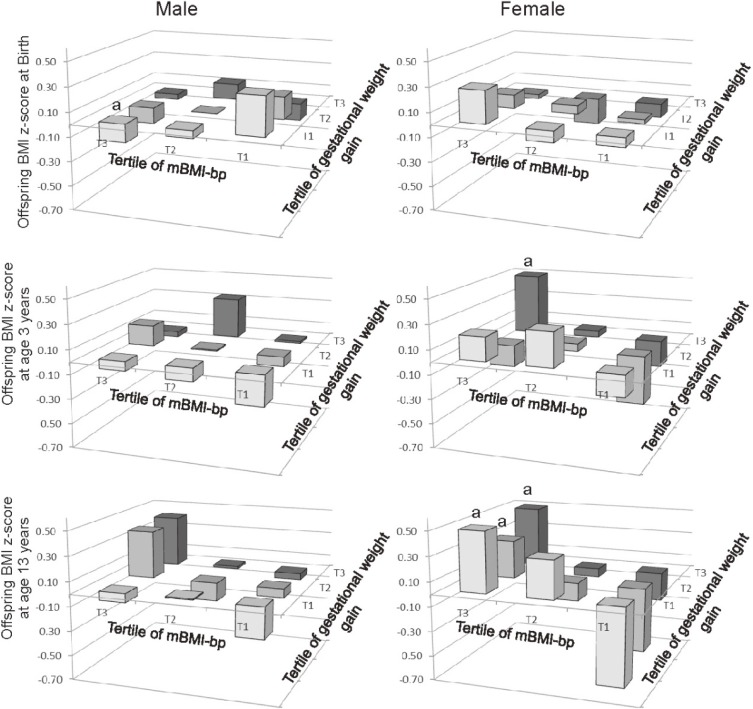
Least square means of offspring BMI z-scores at birth, age 3 years, and age 13 years among nine groups of participants according to tertiles of maternal BMI z score and gestational weight gain. Mean values of offspring BMI z-score were calculated after adjusting for offspring birth weight, gestational age, maternal age at delivery, and smoking during pregnancy. BMI, body mass index; mBMI-bp, maternal BMI z-score at the beginning of pregnancy. ^a^significant changes from lowest (T1) to highest (T3) tertiles of mBMI-bp.

Table 4 shows associations between mBMI-bp and offspring BMI z-scores at birth, at age 3 years, and at age 13 years. In male offspring, mBMI-bp was not associated with offspring BMI z-scores at birth and at age 3 years, while there was a positive association between mBMI-bp and offspring BMI z-score at age 13 years. In female offspring, mBMI-bp was positively associated with offspring BMI z-scores at birth, at ages 3 years, and at age 13 years. The association between mBMI-bp and offspring BMI z-score at age 13 years was stronger than at birth. Figure 1 shows trends of offspring BMI z-scores from the lowest to highest tertiles of mBMI-bp in each tertile of gestational weight gain. In female offspring at age 13 years, trends in every tertile group of gestational weight gain significantly increased from the lowest to highest mBMI-bp groups.

**Table 4.** Relationships between offspring BMI z-score at various ages and mBMI-bp

	Offspring BMI z-score at birth		Offspring BMI z-score at age 3 years		Offspring BMI z-score at age 13 years	
		
	β	*P* value	β	*P* value	β	*P* value
Male						
Model 1	0.006	0.918	0.063	0.310	0.135	0.029
Model 2	0.012	0.848	0.066	0.300	0.154	0.015
Model 3	0.040	0.552	0.102	0.132	0.205	0.002
Model 4	−0.045	0.474	0.075	0.277	0.190	0.005
Female						
Model 1	0.178	0.009	0.225	<0.001	0.468	<0.001
Model 2	0.131	0.033	0.203	0.003	0.491	<0.001
Model 3	0.166	0.011	0.241	0.001	0.509	<0.001
Model 4	0.112	0.014	0.221	0.002	0.508	<0.001

β, standardized regression coefficient; BMI, body mass index; mBMI-bp, maternal BMI z-score at the beginning of pregnancy.

Multiple regression analysis was used to examine relationships between offspring BMI z-score at various ages (dependent variable) and maternal BMI z-score and potential confounders (independent variables).

Model 1: Unadjusted.

Model 2: Adjusted for gestational age, maternal age at delivery, and smoking during pregnancy.

Model 3: Adjusted for factors in Model 2, as well as gestational weight gain.

Model 4: Adjusted for factors in Model 3, as well as offspring birth weight.

## DISCUSSION

This study examined the relationship between maternal BMI at the beginning of pregnancy and offspring BMI at several growth stages in a population-based cohort. The results of this study showed a positive association between mBMI-bp and offspring BMI at age 13 years, and this association was stronger than at birth and at age 3 years. Thus, the relationship between maternal pre-pregnancy weight and offspring weight strengthens as children develop. The association between maternal weight and offspring weight in adolescence might be explained by the extrauterine nutritional environment, which is attributed to maternal lifestyle. Indeed, maternal lifestyle, including dietary habits, has been shown to be strongly associated with offspring lifestyle,^26^ and maternal eating behavior reportedly influences offspring eating behavior.^8^ Thus, offspring weight in adolescence might be largely affected by their mother’s lifestyle. Strategies to improve maternal lifestyle may contribute to the prevention of childhood obesity.

The ABCD study reported that maternal pre-pregnancy BMI was positively associated with offspring BMI at age 5–6 years.^13^ The Nurses’ Health Study II and the Nurses’ Mothers’ Cohort study also reported that obese mothers showed a 6-fold increase in the odds of having obese adolescents, compared to those with a normal body weight.^14^ Moreover, a retrospective cohort study in Ohio reported that childhood obesity at ages 2–4 years was associated with maternal obesity in early pregnancy.^9^ The Jerusalem Perinatal Study also reported that maternal pre-pregnancy BMI was positively associated with offspring BMI at age 32 years, independently of gestational weight gain.^27^ Although results from these previous studies do not conflict with those of the present study, they did not compare the strength of the relationship between maternal weight and offspring weight at several growth stages and did not report temporal changes in the relationship. Moreover, results in the previous studies were based on self-reported maternal weight and maternal height,^10^^,^^11^^,^^13^^,^^14^ whereas the present study used MCH Handbook records, which provide highly reliable baseline data on pre- and post-pregnancy maternal height and weight. Given the tendency to overestimate self-reported height and underestimate self-reported weight,^28^ true maternal BMIs may have been larger than BMIs calculated using self-reported anthropometry. To provide clearer evidence on the association between pre-pregnancy maternal BMI and offspring BMI, accurately recorded anthropometric values are essential. In this regard, the present study used medical records from the MCH Handbook, which contains accurate anthropometric values recorded by medical staff, including obstetricians, pediatricians, public health nurses, and midwives. Thus, the associations between maternal BMI and offspring BMI determined in this study are based on strong evidence in the form of highly accurate data.

This study has some limitations worth noting. First, we used maternal BMI at the beginning of pregnancy, instead of maternal BMI at the time of implantation (pre-pregnancy). However, the prevalence of overweight women at the initial stage of pregnancy in the present study was similar to that of age-matched non-pregnant women in the National Health and Nutrition Survey in Japan.^29^ Therefore, it is likely that the initial stage of (or early) pregnancy nearly matches the timing of implantation (pre-pregnancy). Second, participants were selected from only one city in Japan. Thus, the participants may not be representative of the entire Japanese population. Third, among the 790 students who participated in the health examination, 480 students (approximately 60%) were analyzed in the present study. Parents or guardians transcribed information from the MCH Handbook to a questionnaire of their own free will. MCH Handbook records could not be obtained for 310 students (approximately 40%). This may have led to substantial selection bias. Thus, caution should be exercised when generalizing these results. However, there were no significant differences in characteristics between included and excluded participants (Table 1). Fourth, associations between mBMI-bp and offspring BMI were adjusted for gestational age, maternal age at delivery, offspring birth weight, and smoking during pregnancy, but not for other potential confounders, such as alcohol consumption, parity, socioeconomic status, passive smoking in childhood, breastfeeding, nutrition, physical activities, and exposure to environmental chemicals.^30^ Finally, we performed multiple regression analyses between maternal BMI z-score at the beginning of pregnancy and offspring BMI z-scores at each age separately. Offspring BMI z-scores at each age were not independent. Moreover, as this study examined multiple outcomes (ie, offspring BMI z-scores at birth, at age 3 years, and at age 13 years), multiplicity problems might have arisen in our results.^31^

In conclusion, the present population-based retrospective cohort study suggests that maternal weight is positively associated with offspring weight, and that maternal body weight is more strongly associated with pubertal weight than birth weight or infantile weight. The relationship between maternal pre-pregnancy weight and offspring weight may strengthen as children develop. Therefore, maternal lifestyle may influence offspring weight in adolescence.
